# Marine guanidine alkaloids crambescidins inhibit tumor growth and activate intrinsic apoptotic signaling inducing tumor regression in a colorectal carcinoma zebrafish xenograft model

**DOI:** 10.18632/oncotarget.13068

**Published:** 2016-11-04

**Authors:** María Roel, Juan A. Rubiolo, Jorge Guerra-Varela, Siguara B. L. Silva, Olivier P. Thomas, Pablo Cabezas-Sainz, Laura Sánchez, Rafael López, Luis M. Botana

**Affiliations:** ^1^ Department of Pharmacology, Universidade de Santiago de Compostela, Campus Lugo, 27002 Lugo, Spain; ^2^ Department of Genetics, Universidade de Santiago de Compostela, Campus Lugo, 27002 Lugo, Spain; ^3^ Geoazur, UMR Université Nice Sophia Antipolis-CNRS-IRD-OCA, 06560 Valbonne, France; ^4^ Laboratoire de Pharmacognosie, UMR CNRS 8076 BioCIS, LabEx LERMIT, Université Paris-Sud, Faculté de Pharmacie, 92290 Châtenay-Malabry, France; ^5^ School of Chemistry, Marine Biodiscovery, National University of Ireland Galway, SW4 Galway, Ireland; ^6^ Translational Medical Oncology, Health Research Institute of Santiago (IDIS), Complexo Hospitalario Universitario de Santiago de Compostela (SERGAS), 15706 Santiago de Compostela, Spain

**Keywords:** crambescidins, cell cycle inhibition, apoptosis, zebrafish xenograft model, cancer treatment

## Abstract

The marine environment constitutes an extraordinary resource for the discovery of new therapeutic agents. In the present manuscript we studied the effect of 3 different sponge derived guanidine alkaloids, crambescidine-816, -830, and -800. We show that these compounds strongly inhibit tumor cell proliferation by down-regulating cyclin-dependent kinases 2/6 and cyclins D/A expression while up-regulating the cell cyclin-dependent kinase inhibitors -2A, -2D and -1A. We also show that these guanidine compounds disrupt tumor cell adhesion and cytoskeletal integrity promoting the activation of the intrinsic apoptotic signaling, resulting in loss of mitochondrial membrane potential and concomitant caspase-3 cleavage and activation. The crambescidin 816 anti-tumor effect was fnally assayed in a zebrafish xenotransplantation model confirming its potent antitumor activity against colorectal carcinoma *in vivo*.

Considering these results crambescidins could represent promising natural anticancer agents and therapeutic tools.

## INTRODUCTION

The increasing interest in screening for novel agents from marine organisms is supported by their ability to synthesize an arsenal of bioactive secondary metabolites with pharmacologically significant activities and unusual chemical structures, quite different from those produced by terrestrial ones [1]. Cytarabine (Ara-C) and eribulin mesylate are notable examples of antitumor agents originally extracted from sponges that have been approved as anticancer drugs and are already on the market. Many others have made it to different phases of clinical trials, as for example, LAF389 and KRN7000 [1, 2]. Accordingly, sponges have emerged as an important target for research on new agents of therapeutic interest.

*Crambe crambe* is a marine encrusting sponge of the *Poecilosclerida* order from which several pentacyclic guanidine alkaloids (PGAs) have been isolated and structurally characterized since the first chemical studies were made in the early 90s [3, 4]. Nowadays these PGAs are divided into two families known as crambescins and crambescidins [3].

**Figure 1:**
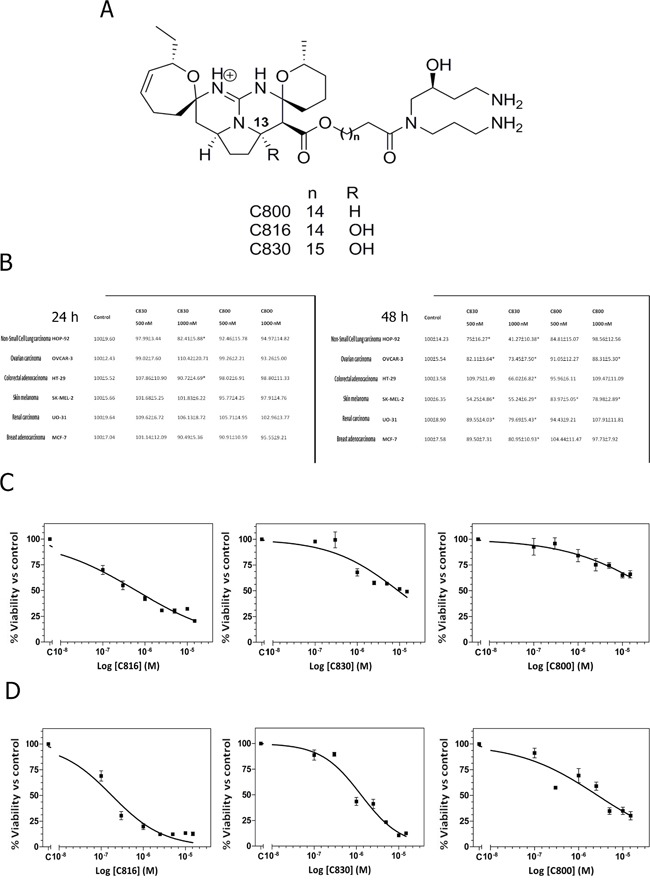
Crambescidins decrease viability of a wide range of tumor cells **A.** Chemical structures of crambescidin 816, crambescidin 830 and crambescidin 800. **B.** Viability of HOP-92, OVCAR-3, HT-29, SK-MEL-2, OU-31 and MCF-7 cells after C830 and C800 treatments for 24 and 48 h, determined by the MTT method. *Significant difference with respect to controls (*p* <0.01). **C.** Dose-dependent decrease of cell viability in response to C816, C830 and C800 determined by the alamarBlue^®^ assay. HepG2 cells were treated with different concentrations of C816, C830 and C800 ranging from 0.1 to 15 μM for 48 h to determine the IC_50_ dose. **D.** Dose-dependent decrease of HepG2 cells viability in response to 0.1-15 μM C816, C830 and C800 and IC_50_ determination at 72 h.

Crambescidins are a family of compounds very similar to the well-known marine alkaloid Ptilomycalin A, more specifically, they are pentacyclic guanidines linked by a linear ω-hydroxy fatty acid to a spermidine or hydroxispermidine unit (Figure 1A) [5]. Several of these molecules have been patented due to their cytotoxic, antifungal and antiviral activities and some of them, as for example crambescidin 816 (C816), also have recognized effects over calcium ion channels [3, 5, 6]. Furthermore, in 2007 crambescidin 800 (C800) was also proposed as an antioxidant agent against hypoxia, nitric oxide and glutamate-induced oxidative stress [7]. Other previously reported biological activities of C800 include its ability to combat liver and blood stage of *Plasmodium spp*. [8] and to induce erythroid differentiation of chronic human myelogenous leukemia cells [9].

While some previous studies revealed the cytotoxicity of C816 against human colon carcinoma cells (IC_50_ 0.24 μg/ml) [3] and others inquired the relationship between tumor selectivity and crambescidin alkaloids analogs structures [10], the understanding of the underlying cellular mechanisms responsible for their antitumor activities is scarce up to date. In this context, we previously assayed the transcriptomic alterations induced by C816 on the human liver carcinoma cell line HepG2 showing that this compound arrests cell cycle progression disrupting cell-cell and cell-matrix adhesions [11].

Encouraged by these interesting findings we decided to further investigate C816 and other side chain analogs of the crambescidin family (C800 and C830) in order to elucidate the effects of each tested compound against a wide range of human-tumor-derived cell lines and their relative potency.

We also aim to determine whether these three PGAs might lead to tumor cell death through the same molecular pathways and to further characterize their mechanisms of action. For these purposes, crambescidins activities against cancer cells were assayed *in vitro* and C816 antitumor efficacy ultimately tested *in vivo* using a zebrafish xenograft model.

During the last decade the zebrafish (*Danio rerio*) has rapidly become a popular model organism in many areas of biomedical research since it presents several major advantages compared to rodents. The growing interest of both, adult and embryo stage zebrafish, in pharmacology is supported by several hallmarks of this small vertebrate as its fecundity, fast development, big offspring size, transparency, high genetic and physiological homology to humans and theease of genetic manipulation. These characteristics provide important utilities and opportunities to accelerate the process of drug discovery by contributing to disease modeling, assay of toxicity, bioavailability evaluation and lead molecules identification. Previous works have carefully evaluated the pros and cons of zebrafish cancer models and validated this approach [12–14]. In fact, zebrafish xenografts have been widely used before as reliable whole-animal model systems to rapidly screen for small-molecule drug candidates [15–17].

## RESULTS

### Crambescidins reduce viability of tumor cells

C830 and C800 effects on tumor cell proliferation and survival were assayed by the MTT method. C816 (Figure 1A) cytotoxicity against a wide range of tumor cells has been previously established (17). Based on these results, concentrations of 0.5 μM and 1 μM were selected in order to facilitate any possible comparison with existing data. Results showed that after 24 h just C830 decreased cell viability of lung non-small cancer cells (HOP-92) and colon carcinoma (HT-29) cells while, in the case of C800, no effect was observed (Figure 1B). Nevertheless, after 48 h either C830 or C800 decreased tumor cells survival. C830 significantly decreased cell viability of all tested cell lines at the two concentrations assayed (Figure 1B). Cytotoxic activity of C800 was just significant against melanoma (SK-MEL-2) and ovarian carcinoma (OVCAR-3) cell lines (Figure 1B). Moreover, cell viability reduction caused by C830 was higher when compared with results obtained for C800 and lower when compared with those previously obtained for C816 (17). To quantify the effect produced by each of these compounds and to establish and compare their potency, IC_50_ values against HepG2 cells were calculated.

The *in vitro* IC_50_ values of C816 and C830 after 48 h of treatment were 0.57 μM and 9.73 μM, respectively (95% confidence intervals 0.41 μM-0.79 μM and 5.84 μM-16.22 μM) (Figure 1C). Although at the same time-point C800 significantly reduced tumor cell viability, the percentage of the maximum effect did not reach 50% (Figure 1C). Considering this, the IC_50_ after long-term treatments (72 h) was also determined for each of the three compounds. The IC_50_ values (95 % confidence limits) of C816, C830 and C800 were 0.18 μM (0.12 μM-0.25 μM), 2.11 μM (1.48 μM-3.00 μM) and 2.66 μM (1.77 μM-4.13 μM), respectively (Figure 1D).

### Crambescidins 800 and 830 disrupt cellular adhesion and cytoskeletal integrity

Loss of cell attachment is one of the early effects of C816 on tumor cells. Alterations of focal adhesion plaques, tight junctions and cytoskeleton proteins start after short periods of exposure (6 h) to low concentrations (0.5 and 1 μM) of C816 and progress up to 24 h (17). To elucidate if cell detachment was just caused by C816 or could be a broad effect of the crambescidin family of compounds, we assayed the effect of C830 and C800 on cell adhesion and cytoskeleton integrity. Considering the IC_50_values previously obtained, a concentration of 2.5 μM was selected in order to assure the adequate conditions to detect any possible alteration caused by these molecules. As a benchmark for comparison, treatments with 2.5 μM C816 were also included.

**Figure 2:**
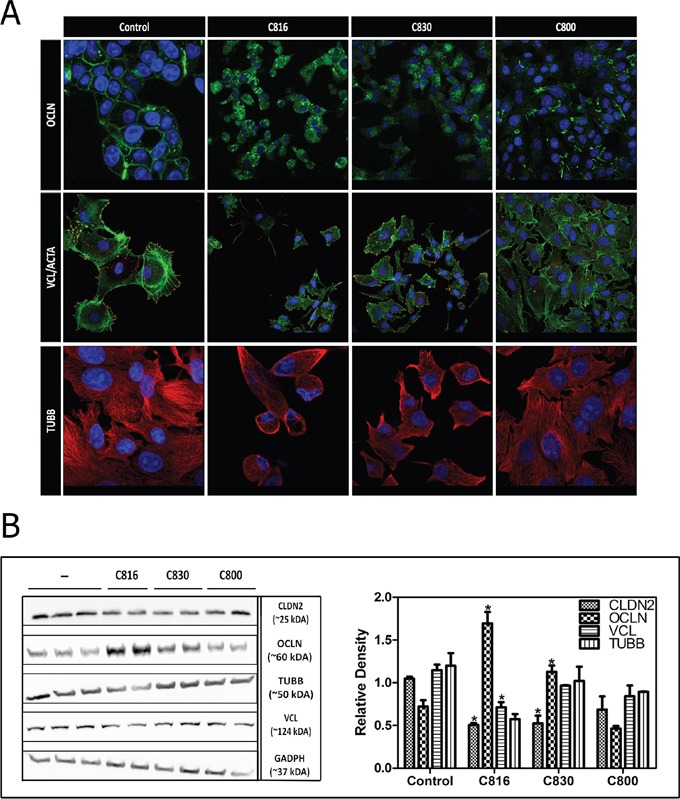
Crambescidins alter cell adherence and cytoskeletal integrity of tumor cells **A.** OCLN (green), VCL (red), F-ACTA (green) and β-TUBB (red) detection by confocal microscopy in control and 2.5 μM C816, C830 and C800-treated cells after 24 h. Colocalization of F-ACTA and VCL is shown in yellow and representative images of control and treated cells are shown. Hoechst 33258 was included for nuclei counterstaining (blue). **B.** Left: Determination of soluble CLDN2, OCLN, TUBB, VLC and GADPH levels. HepG2 cells were treated with 2.5 μM C816, C830 and C800 for 24 h and then soluble protein fractions obtained from cell lysates of treated and untreated cells were analyzed by western blot. Right: Quantification of the differences in protein levels among control and 2.5 μM C816, C830 and C800 treated cells.* Significant differences respect to controls (*p* <0.05).

After 24 h both, C830 and C816, produced a remarkable occludin (OCLN) translocation from cell membrane to the cytoplasm. In the case of C800 a slight increase in occluding cytoplasmic levels was also observed (Figure 2A). Although all the crambescidins induced the internalization of OCLN from cell membrane, results showed that the translocation produced by C816 was higher when compared with that produced by C830 and C800. Similarly, changes in OCLN localization produced by C830 were more notable than the alterations caused by C800.

Crambescidins also reduced tumor cells adhesion by decreasing vinculin-containing adhesion plaques leading to reduction of cell-substratum interactions (Figure 2A). Cytoskeleton alterations induced by C816 and C830 were due to tubulin depolymerization and disassembly of actin stress fibers (Figure 2A). Once again, observed effects were more apparent for C816 followed by C830 and C800, respectively. Crambescidins effect on tubulin polymerization was also assayed. These compounds partially inhibited or delayed tubulin polymerization being C816 the most potent of the three PGAs (Supplementary Figure S2A, S2B).

To quantify the effect of each compound on tight junctions and focal adhesions proteins levels, western blot analysis of claudin-2 (CLDN2), OCLN and vinculin (VCL) were performed. C816 treatments were again included for comparison purposes and as positive controls. Results supported those previously obtained by confocal microscopy confirming that C816 and C830 are capable of decreasing CLDN2 and increasing OCLN cytoplasmic levels (Figure 2B). OCLN localized at tight junctions was lost after centrifugation as just the soluble protein fraction was recovered for western blot. Finally, no significant variations in tubulin-β (TUBB) levels were detected and just C816 treatments significantly decreased VCL (2B).

### Crambescidins affect CDKs and cyclins transcriptional regulation leading to G1 cell cycle arrest

**Figure 3:**
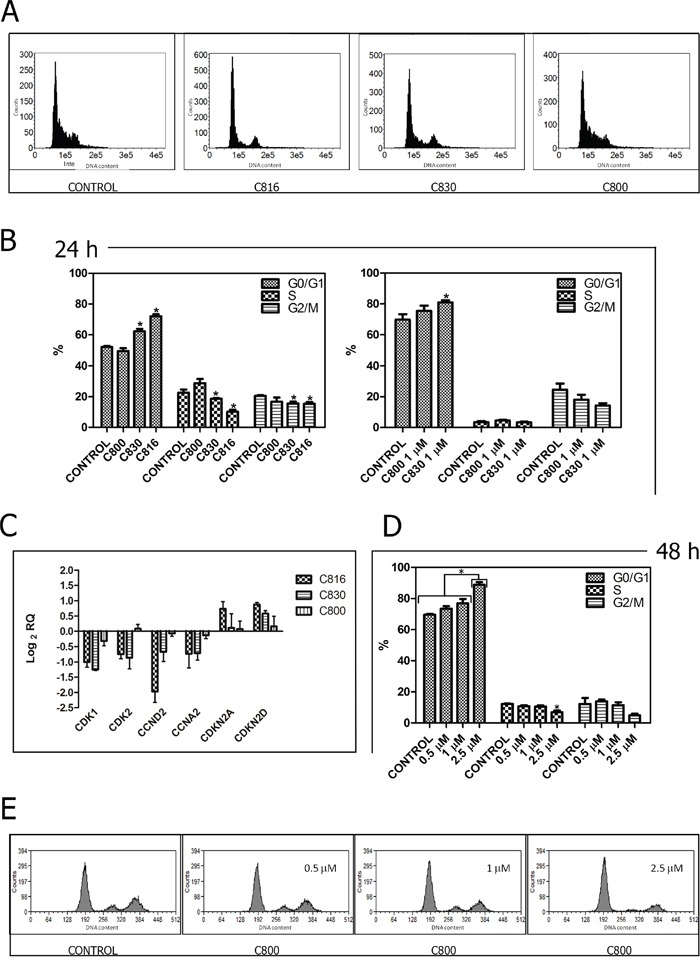
Crambescidins arrest cell cycle progression in the G0/G1 phase **A.** Representative histograms of the cell cycle obtained after flow cytometry analysis of 2.5 μM C816, C830 and C800-treated HepG2 cells for 24 h. **B.** Quantification of the cell population percentages in each phase of the cell cycle in control and HepG2 cells treated with 2.5 μM C816, C830, C800 and 1 μM C830 and C800 for 24 h (*p* <0.05, n =2). **C.** Down-regulation of CDK1, CDK2, CCND2 and CCNA2 mRNA and up-regulation of CDKN2A and CDKN2D mRNA in 2.5 μM C816, C830 and C800-treated cells after 24 h as determined by qRT-PCR. **D.** Quantification of the cell population percentages in each phase of the cell cycle in control and HepG2 cells treated with 0.5, 1 and 2.5 μM C800 for 48 h (*p* <0.05, n =2). **E.** Representative histograms of the cell cycle obtained after flow cytometry analysis of 0.5, 1 and 2.5 μM C800-treated cells for 48 h.

Considering their capacity to inhibit tumor cell growth, C830 and C800 effect on cell cycle progression was evaluated. Results showed that after 24 h, 1 μM and 2.5 μM C830 caused an accumulation of HepG2 cells in the GO/G1 phase. At this time-point no restriction was produced by C800 (Figure 3A, 3B). Analysis of cell cycle regulators expression showed that C830 down-regulated cyclins A and D (CCND2 and CCNA2), and cyclin-dependent kinases 2, 6 and 1 (CDK2, CDK6 and 1). On the contrary, this compound up-regulated cyclin-dependent kinase inhibitors 1 and 2 (CDKN2A, CDKN2D and CDKN1A) (Figure 3C). In the case of C800, no variations were detected after 24 h (Figure 3C). In fact, cell cycle inhibition produced by C800 was just observed after 48 h of treatment (Figure 3D, 3E).

### Crambescidins induce mitochondrial-mediated apoptosis of HepG2 cells

**Figure 4:**
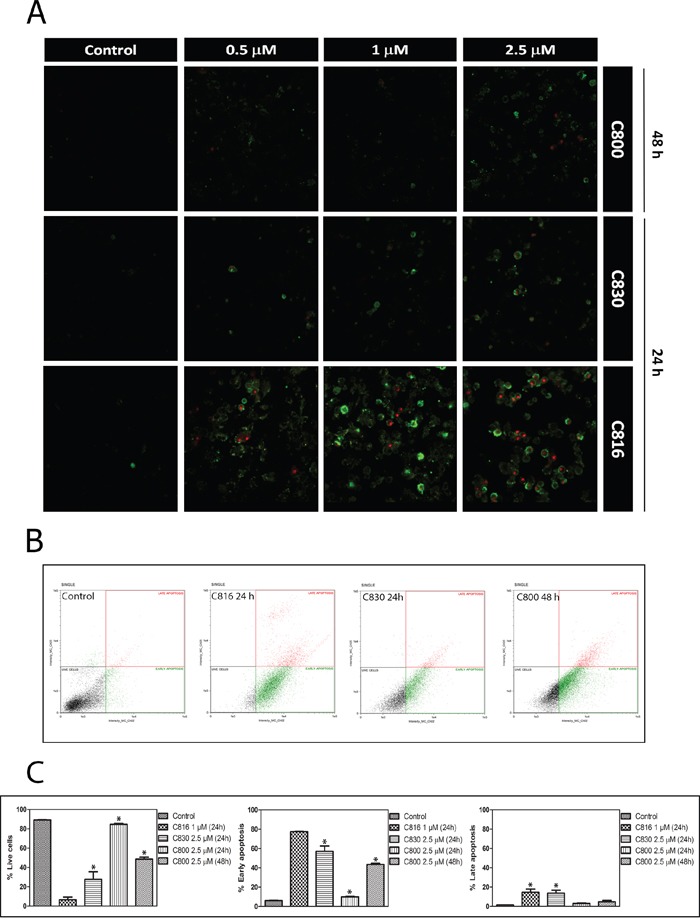
Crambescidins induce intrinsic apoptotic death of tumor cells **A.** Annexin V and PI staining of HepG2 cells after 0.5, 1 and 2.5 μM C816, C830 and C800 treatments for 24 and 48 h analyzed by confocal microscopy. **B.** Annexin V apoptosis determination of HepG2 cells treated with 1 μM C816, 2.5 μM C830 and 2.5 μM C800 for 24 h and 2.5 μM C800 for 48 h. Representative plots. **C.** Quantification of the different subpopulations as determined by flow cytometry.

**Figure 5:**
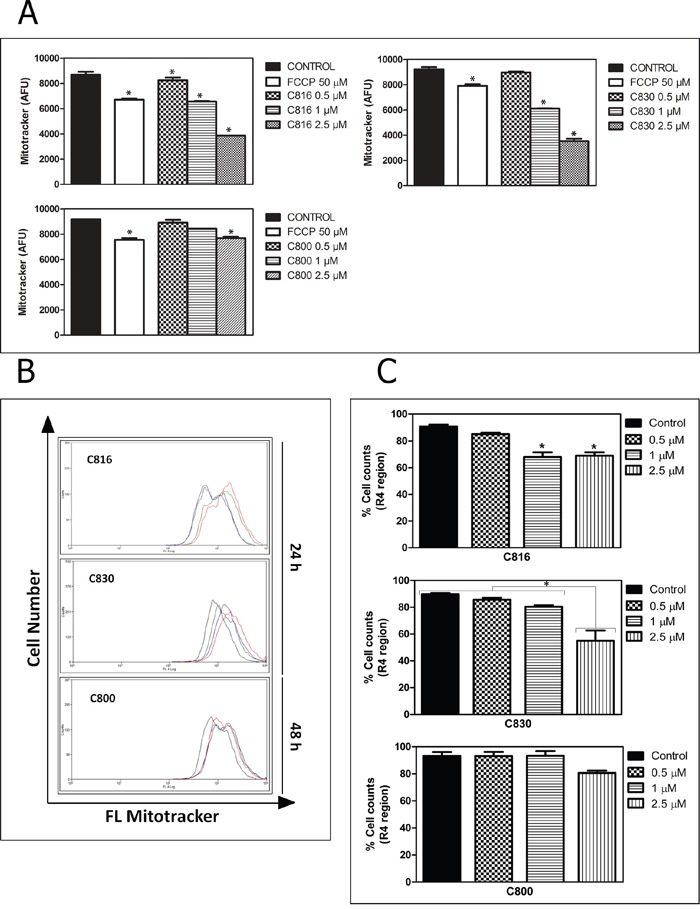
Crambescidins' effect on mitochondrial function **A.** Effects of 0.5, 1 and 2.5 μM C816, C830 and 0.5, 1 and 2.5 μM C800 treatments on mitochondrial membrane potential after 24 and 48 h, respectively as determined using MitoTracker^®^ Red CM-H2XRos staining (*p*<0.05). **B.** Representative overlay histograms of mitochondrial viability of control (red line), 0.5 (green line), 1 (blue line) and 2.5 μM (black line) treated cells as determined by flow cytometry using Mito Tracker^®^ Deep Red-FM. **C.** Population percentages of HepG2 cells (mean ± SEM) in the R regions (for R1, 2, 3 and 4 regions determination see Supplementary Figure S1) after 24 h treatments with C816, C830 and 48 h with C800 (*p* <0.05).

**Figure 6:**
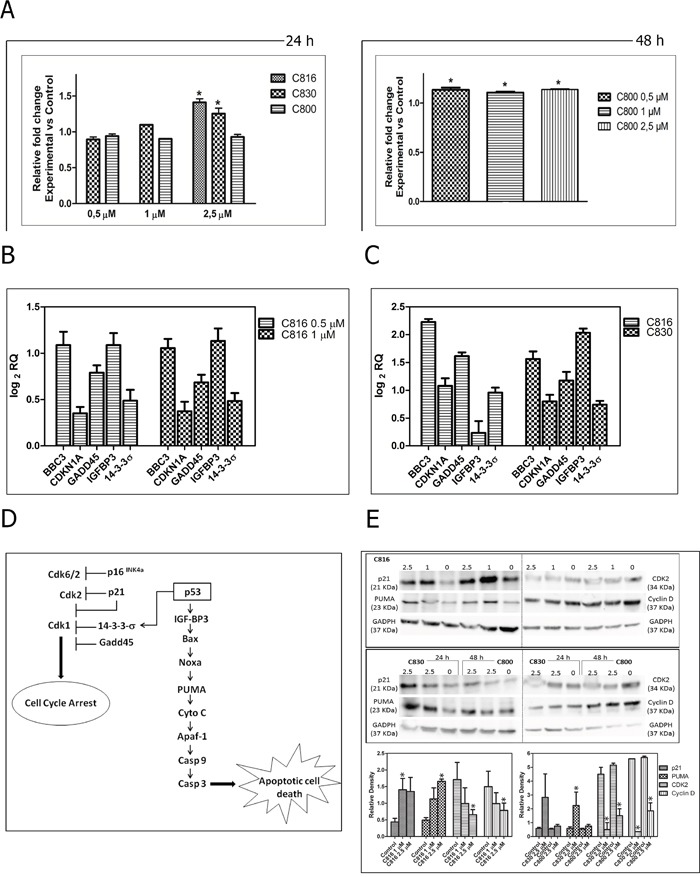
Death signaling and effector pathways activated by crambescidins **A.** Caspase-3 activity in HepG2 cells treated with C816 and C830 for 24 h and with C800 for 48 h. *Significant differences with respect to controls (*p* <0.05, n = 3). **B.** Up-regulation of BBC3, CDKN1A, GADD45, IGFBP3 and 14-3-3σ genes mRNA in C816-treated cells for 24 h as determined by qRT-PCR. **C.** Up-regulation of BBC3, CDKN1A, GADD45, IGFBP3 and 14-3-3σ genes mRNA in 2.5 μM C816 and C830-treated cells for 24 h as determined by qRT-PCR. **D.** Schematic illustration of the molecular downstream targets of the cellular response elicited by crambescidins that mediate their different biological outcomes. **E.** Western blot analysis of proteins involved in crambescidin-mediated cell cycle arrest (p21, Cdk2 and cyclin D) and apoptosis (PUMA) induction. * Significant differences respect to controls (*p* <0.05).

C830 and C800 ability to activate programmed cell death following cell cycle arrest was assayed by different methods. Flow cytometric and confocal microscopy analysis using Annexin V and IP showed that C830 induces cellular apoptosis after 24 h. C800 also increased the apoptotic population but after 48 h (Figure 4A, 4B, 4C). Phosphatidylserine translocation caused by C816 was higher than that produced by C830 and C800 (Figure 4A, 4B, 4C). Mitochondrial function and integrity were also analyzed since these organelles play a major role in mediating the majority of apoptotic pathways in mammalian cells [18, 19]. After 24 h, C816 and C830 disrupted mitochondrial integrity with a dose-dependent effect (Figure 5A, 5B, 5C). Mitochondrial integrity alterations caused by 2.5 μM C800 were also observed after 48 h treatments (Figure 5A, 5B, 5C). Since apoptosis is primarily mediated through activation of specific intracellular cysteine proteases known as caspases [20], activity of the central apoptotic effector enzyme caspase-3 was measured. Fluorometric analyses revealed a significant increment in caspase-3 activity caused by C830 within 24 h. C800 also increased capase-3 activity but after 48 h treatments (Figure 6A).

### Crambescidins activate some key downstream events of the p-53 pathway

C816 and C830 up-regulated several downstream targets of the p53 transcription factor that mediate different biological outcomes (Figure 6B, 6C). Both, C830 and C816, increased the expression of IGF-BP3 and PUMA (BBC3) genes, both directly activated by p53 as part of the program to bring about apoptosis (Figure 6C). These compounds also induced the expression of several genes of the p53 network implicated in promoting cell cycle arrest (Figure 6D). These changes were also observed at the protein level as determined by the increase of CDKN1A and PUMA and the decrease CDK2 and cyclin D detected by Western Blot analysis (Figure 6E).

### Crambescidins can induce apoptotic cell death via p-53-independent mechanisms

**Figure 7:**
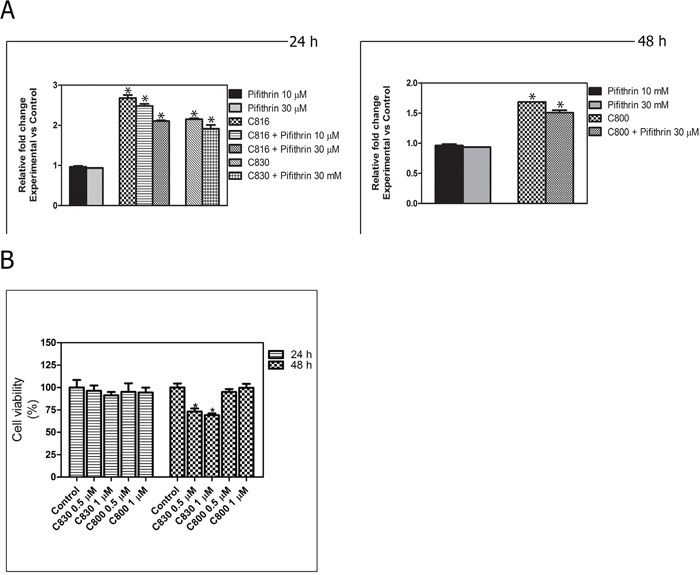
p-53 implication in crambescidin-induced apoptosis **A.** Caspase-3 activity determination for C816, C830 and C800-treated cells after p-53 inhibition. *Significant differences with respect to controls and between treatments groups (*p* <0.01). **B.** Viability of p53-null PC3 cells after C830 and C800 treatments during 24 and 48 h as determined by the MTT method. *Significant difference with respect to controls (*p* <0.01).

To elucidate if crambescidins could induce cell cycle arrest and apoptotic death of tumor cells in a p53-independent manner, casapase-3 activity determination following p53 inhibition with Pifithrin α in p53 expressing cells, and effects on the p53-null PC3 prostate cancer cells viability were also assayed. Statistically significant decreases of caspase-3 activity between functional and p-53 inhibited cells were detected for the three PGAS tested (Figure 7A). Even this, p53 inhibition could not completely abolish apoptosis induced by crambescidins. Similarly, low concentrations of C830 reduced PC3 cells growth by approximately 30% after 48 h while no significant induction of cell death was caused by the same concentrations of C800 (Figure 7B).

### Crambescidin 816 impairs growth of colon carcinoma xenograft models

Three doses of C816 (0.5, 1 μM, and 2 μM) were selected to assay the *in vivo* antitumor activity of C816 based on toxicity assays results. Experiments were carried out at 35°C to favor tumor cell growth. In addition, embryo survival rates were annotated at the end of the experiments, being the obtained percentages 53.23 % (33 out of 62) for negative controls, 28.17 % (20 out of 71) for injected embryos without treatment, 38.64 % (17 out of 44) for 0.5 μM C816, 42.50 % (17 out of 40) for 1.0 μM C816, 52.00 % (13 out of 25) for 2.0 μM C816 and 50.00 % (20 out of 40) for 500 μM 5-FU.

**Figure 8:**
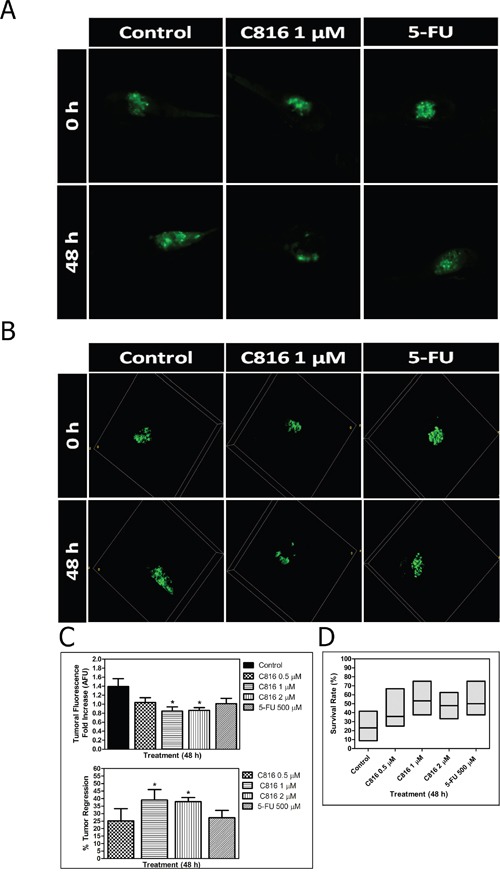
Crambescidin 816 induces tumor regression in an *in vivo* colorectal carcinoma model **A.** *In vivo* anticancer activity of C816 in a human tumor xenograft model. Representative Z-projection images of the tumor development in control and treated embryos after 48 h. **B.** Three-dimensional reconstruction of tumor development in control and treated embryos after 48 h. **C.** Tumor fluorescence intensities (mean ± SEM) and percentages of decrease in tumor volumes of treated embryos after 48 h. **D.** Survival rates of control and treated embryos (median and Q_1_, Q_3_). *Significant difference with respect to controls (*p* <0.05).

The tumor growth in embryos treated with C816, 5-FU and untreated controls was observed after completion of treatments. Embryos treated with 0.5 μM C816 showed a slight tumor regression while, 1 and 2 μM C816 caused a significant decrease of tumor growth compared to untreated controls (p < 0.05) (Figure 8A, 8B, 8C). Administration of 500 μM 5-FU also reduced tumor growth but without reaching the effect produced by C816 (Figure 8A, 8B, 8C). Although not significant, a slight increase in C816-treated embryos survival rates was also detected (Figure 8D).

## DISCUSSION AND CONCLUSIONS

We have previously reported C816 ability to reduce viability of a wide range of human tumor-derived cell lines also inhibiting cell migration and adhesion [11]. Herein, we further investigated the molecular mechanisms underlying these bioactivities and focused on other members of the crambescidin family of compounds.

Results showed that either C830 or C800 reduce tumor cells viability mainly after 48h exposure to low concentrations of these molecules (0.5 μM and 1 μM). However, the inhibition percentages were always lower than that produced by the same concentrations of C816. The IC_50_ values of the three compounds supported these results confirming that C816 reduces tumor cells survival with a higher potency than C830 and C800.

In the present study we also show that crambescidins decreased cell-cell and cell-matrix adhesions. While disruption of tight junctions and focal adhesions appears early after 6 h exposure to low concentrations of C816 and progress up to 24 h, in the case of C830 higher concentrations (2.5 μM) and longer treatments (24 h) were necessary to produce similar effects. Alterations produced by 2.5 μM C800 were less noticeable and just comparable with those produced by 0.5 μM C816 after 6 h [11]. Accordingly, immunocytochemistry and western blot analysis showed that crambescidins decrease the concentrations of two major integral proteins (OCLN and CLDN2) in the plasma membrane disrupting tight junctions by inducing OCLN cytoplasmic translocation and reducing CLDN2 protein expression. Furthermore, C816 and C830 disrupt cell attachment to extracellular matrix through the reduction of VCL in focal adhesions. Both compounds also induce tubulin depolymerization and stress fibers disassembly compromising cytoskeletal integrity and dynamics. A direct effect of crambescidins on tubulin polymerization was also observed. Both C816 and C830 partially inhibited tubulin polymerization while C800 only produced a temporal delay reaching control levels after 1 h. Alterations of the tubulin cytoskeleton were not observed after 24 h treatments with 2.5 μM C800 indicating that, for this compound, cytoskeletal rearrangements might be later events following cell detachment [21, 22].

Crambescidins inhibited the cell cycle progression in the G0/G1 phase with different potencies (C816>C830>C800). In direct relation with this observation, the assayed compounds down-regulated CCND2, CCNA2, CDK2, CDK6 and CDK1, as well as up-regulated CDKN2A, CDKN2D and CDKN1A. These changes ultimately resulted in the observed G0/G1 cell cycle blockage since CDK2 associated with cyclins A, D and E, and also cyclin D in association with CDK4 or CDK6, control the G1 to S phase transition [23, 24]. Similarly, CDKN2A, CDKN2D and CDKN1A cyclin-dependent kinase inhibitors prevent S-phase entry [25]. Crambescidins up-regulated different genes of the p53 transcriptional program resulting in cell cycle arrest and apoptosis. In fact, p-53 ability to promote cell cycle arrest is fairly well understood in terms of its ability to transactivate CDKN1A, GADD45 and 14-3-3 genes [26–29], all of which were induced by C816 and C830 after 24 h. Other target genes induced by crambescidins are known to be downstream mediators of apoptotic cell death [30–34]. Thus, C816 and C830 increased the expression of IGFBP3 and other p-53 activated genes (BAX, NOXA, and PUMA) involved in mitochondrial damage with concomitant release of cytochrome *c* into the cytoplasm [35]. These results are in concordance with the disruption of the mitochondrial integrity caused by the three crambescidins and with the subsequent activation of the executor caspase-3 observed.

The influence of the p53 response in deciding tumor cell fate after crambescidin exposure was also confirmed. Caspase-3 activity was significantly decreased following p-53 inhibition and exposure to the three PGAS tested without reaching control level. Furthermore, PC3 cells underwent cell death in response to C830 and C816. Both results indicate that, while the p-53 network can contribute to programmed cell death induction, it is not the unique route involved in its signaling. We further studied the role of p-53 in crambescidins-induced cell death and demonstrated that these compounds increase the transcriptional expression of several components of the p-53 pathway and their respective proteins. Inhibition of p-53 ameliorated but did not abolish crambescidin-induced cell death demonstrating that this tumor suppressor contributes but it is not essential for their effects.

Treatments with low concentrations of C816 induced cell detachment from the ECM within the first 6 hours of exposure in the absence of caspase activation [11]. A similar delay was also observed when cells were treated with high concentrations of C800 for 24 h. The existing interdependences between matrix adhesions, cytoskeleton and signaling pathways regulating cell proliferation and death indicate that transmembrane crosstalks interruption can restrict cell cycle progression through G1 into S phase and lead to anoikis through mitochondrial apoptotic pathways [21, 36–41]. Although in the case of crambescidins obtained results provide strong evidences supporting this possibility, further research is still needed in order to confirm this.

Taken together, these results highlight the existing differences between C816, C830 and C800 in terms of potency, but also support the similarity regarding to their bioactivities and mechanisms of action. The presence of a hydroxyl group at C-13 induces higher biological effects. The hemiaminal group at C-13 is highly reactive and can lead to an iminium after an easy dehydration process. These iminiums are known to be highly electrophilic towards all types of nucleophiles and could explain the higher potency of C816.

Xenograft assays demonstrated that C816 impairs the growth of colorectal carcinoma (CRC) HTC-116 cells *in vivo*. This cell line harbors one of the most common mutations in CRC, a *K*-RAS glycine-to aspartate mutation at codon 13 (Gly^13^Asp), accounting to ~20% of *K-RAS* mutations [42]. Metastatic CRC response to anti-epidermal growth factor receptor antibody therapies inversely correlates with oncogenic activation of the RAS/RAF signaling pathway [43]. Therefore, C816 ability to reduce survival of cancer cells carrying constitutively active RAS and WT *K*-RAS (HT29) is also interesting in terms of its possible therapeutic implications. The higher potency of C816 compared to 5-FU against *K*-RAS mutant cells was also remarkable since 5-FU is commonly used as a standard first or second-line therapy for metastatic CRC.

In conclusion, these results support the interest of crambescidins not just as possible lead drug candidates but also as useful therapeutic tools for the study of cell adhesion remodeling considering the importance of this process as a crucial step in a series of changes that a tumor cell undergoes during malignant transformation.

## MATERIALS AND METHODS

### Reagents and solutions

Eagle's Minimal Essential Medium (E-MEM) and Roswell Park Memorial Institute Medium (RPMI) were purchased from Gibco (Thermo Fisher Scientific Corporation, Madrid, Spain). Fetal bovine serum (FBS) was from Cambrex Corporation (Charles City, USA). Penicillin, streptomycin, 3-[4,5-dimethylthiazol-2-yl]-2,5-diphenyltetrazolium bromide (MTT), anti-β-tubulin, anti-rabbit and anti-mouse IgG horseradish peroxidase-linked species-specific whole antibodies, CY3-conjugated anti-mouse secondary antibody, Hoechst 33258 and propidium iodide were purchased from Sigma-Aldrich (Madrid, Spain). Aurum™ Total RNA Mini Kit, Precision Plus Protein™ Standards Kaleidoscope™ and iTaq™ Universal SYBR^®^ Green Supermix were obtained from Bio-Rad Laboratories (Barcelona, Spain). Polyvinylidene fluoride (PVDF) membranes, anti-actin and anti-vinculin antibodies were purchased from Merck-Millipore (Temecula, USA). Anti-claudin 2 antibody was from Santa Cruz Biotechnology (Dallas, USA). Anti-occludin, anti-occludin Alexa Fluor^®^ 488 conjugate antibodies and Oregon Green^®^ 514 Phalloidin were from Molecular Probes^®^ (Thermo Fisher Scientific Corporation, Madrid, Spain). Anti-p21 and anti-PUMA antibodies were purchased from Cell Signaling while anti-CDK2 and anti-CyclinD antibodies were obtained from Santa Cruz biotechnologies. SuperSignal^®^ West Pico, SuperSignal^®^ West Femto, alamarBlue^®^ dye, oligo-dT, RevertAid™ M-MuLV reverse transcriptase and EnzChek^®^ Caspase-3 Assay kit were obtained from Thermo Fisher Scientific Corporation (Madrid, Spain). Primers were purchased from Integrated DNA Technologies (Iowa, USA) and sequences are available from the authors upon request.

### Crambescidins isolation

Crambescidin 816, crambescidin 830 and crambescidin 800 were purified from *C. crambe* sponges following a previously described protocol [44]. Compounds were then identified by both UPLC-HRMS and NMR analysis (95% purity) and dissolved in DMSO. Treated cells were always exposed to a final DMSO concentration lower than 0.2%.

### Cell culture

HepG2 cell line was purchased from the American Type Culture Collection (ATCC) and cultured in E-MEM supplemented with 10% fetal bovine serum, 100 UI/ml penicillin and 0,1 mg/ml streptomycin. SK-MEL, OU-31, PC-3, OVCAR, MCF-7, HT-29 and HOP-92 cell lines were obtained from the US National Cancer Institute (NCI) and cultured in RPMI medium supplemented with 10% fetal bovine serum, 50 UI/ml penicillin and 0.05 mg/ml streptomycin. All cell lines were maintained at 37°C in a humidified 5% CO_2_ atmosphere.

### Cell viability assays

Cell viability was determined using the MTT method. Briefly, SK-MEL, OU-31, PC-3, OVCAR, MCF-7, HT-29 and HOP-92 cells were seeded in 96-well plates at a density of 8000 cells per well and treated with 0.5 μM C830 and C800 for 24 h. MTT was added to culture medium four hours before incubations ended. Finally, reduced MTT was dissolved in DMSO and absorbance was determined at 570 and 670 nm (reference) using a Bio-Tek Synergy plate reader (Bio-Tek). For each cell line three experiments with *n* = 8 were performed.

### IC_50_ determination

HepG2 cells were seeded in 96 well plates at a density of 8000 cells/well and exposed to 0.1 μM, 0.3 μM, 1 μM, 2.5 μM, 5 μM, 10 μM and 15 μM C816, C830 and C800 for 48 and 72h. Cell viability was determined using the alamarBlue^®^ dye following manufacturer's instructions. Fluorescence values were monitored every two hours at 530 excitation and 590 nm emission wavelengths. IC_50_ determinations were achieved via nonlinear regression analysis of the obtained results using the GraphPad^®^ software. Three experiments with n = 8 were performed.

### Cell cycle analysis

HepG2 cells were treated with 0.5, 1 and 2.5 μM C830 for 24 h and with the same concentrations of C800 for 24 and 48 h. After detachment cells from either control or treated cultures were washed twice in PBS supplemented with 0.1% BSA and 0.3 mM EDTA and fixed in cold 70% ethanol at 4°C for 30 min. Fixed cells were washed twice with PBS before final pellet collection and nuclei staining for 1 h in 50 μL of Telford reagent (75 μM IP, 0.1 mM EDTA, 1.34 mg RNase, 0.1% Triton X-100) at room temperature protected from the light.

Samples were processed using a FACSCalibur™ cytometer (Becton Dickinson) and post-acquisition analysis was carried out using the Summit software (DAKO). Each condition was tested in triplicate and for every replicate a total number of 10 000 events were acquired. Two experiments were performed.

### Cell adhesion and cytoskeletal integrity assessment by confocal microscopy

HepG2 cells were cultured on poly-lysine coated cover slips and treated with 2.5 μM C830, C800 and C816 for 24 h. Cells were fixed with 4% paraformaldehyde for 15 min at 4°C and permeabilized with a solution of 0.2% Triton X-100 in PBS or fixed with 100% methanol for 5 min at −20°C depending on the type of staining. Before labeling, three washes with PBS-0.1% Tween^®^ 20 were made. Cover slips were then incubated with 1:300 anti-occludin (OCLN), anti-vinculin (VCL) and anti-β-tubulin (TUBB) antibodies dissolved in a solution of 2% BSA in PBS for 2 h at room temperature. After washing, cells were incubated again with 1:500 CY3-conjugated anti-mouse secondary antibody for 1 h protected from light. For occludin staining an antibody conjugated with Alexa Fluor^®^ 488 was used avoiding this last step. To visualize F-actin phalloidin conjugated with Oregon Green^®^514 was used. Before mounting, cover slips were rinsed with PBS-0.1% Tween^®^ 20 and 1 μM Hoechst 33258 was added for nuclei counterstaining.

Images were acquired with a NIKON TE2000-3 confocal microscope. Representative images of each condition are presented in this work.

### Tubulin polymerization assay

The effect of crambescidins on tubulin polymerization was assayed using the fluorescence based Tubulin Polymerization Assay Kit (Cytosqueleton) following the manufacturer's instructions. Briefly, a mix of tubulin buffer, 1 mM GTP and 1.9 mg/ml tubulin was added to a 96 well plate previously loaded with the compounds. Tubulin polymerization was immediately monitored for 1 h with excitation and emission wavelengths set at 350 and 430 nm, respectively using a Bio-Tek Synergy plate reader thermostated at 37°C.

### Western blot analysis

Control and treated cells were re-suspended in RIPA lysis buffer (150 mM NaCl, 1% Triton X-100, 0.5% sodium deoxycholate, 0.1% SDS and 50 mM Tris, pH = 8) and kept on ice for 30 minutes. Lysates were then centrifuged at 14000 RPM, 4°C for 15 min and pellets were discarded. Total protein concentrations of samples were quantified using a Direct Detect™ spectrometer (Merk-Millipore) and equivalent protein amounts were resolved by SDS-PAGE and transferred to PVDF membranes.

PVDF membranes were blocked overnight at 4°C in a solution of 3% non-fat milk and then incubated with anti-OCLN 1:3000, anti-VCL 1:5000, anti-claudin 2 (CLDN2), anti-glyceraldehyde-3-phosphate dehydrogenase (GADPH) 2:1000, anti-CDK2 1:1000, anti-cyclin D 1:1000, anti-p21 1:1000 and anti-PUMA 1:1000 antibodies for 3 h at room temperature. After washing (3 X with PBS-0.1% Tween^®^ 20) membranes were incubated with secondary anti-rabbit and anti-mouse IgG horseradish peroxidase-linked antibodies 1:5000 (1 h, room temperature). Finally, membranes were washed and revealed with SuperSignal^®^ West Pico or SuperSignal^®^ West Femto using a Diversity detector (Syngene). Protein quantification was normalized with GADPH and differences between treatments were determined with the image analysis software GeneTools (Syngene).

### Annexin V and propidium iodide staining and analysis

Cells were cultured on poly-lysine coated cover slips at a density of 400 000 cells/well and on 6 well plates until 70-80 % confluency. Cultures were then treated with C830, C800 and C816. After treatment cells were stained with Anexin V and IP using the Annexin V-FITC. Apoptosis Detection Kit (Immunostep) following the manufacturer's instructions. Stained cells were analyzed with a NIKON-TE2000-3 confocal microscope and by flow cytometry with an ImageStream II cytometer (Amnis) (Supplementary Figure S2A). Each condition was tested in duplicate and three experiments were done. Representative images of each condition are presented in this work.

### Mitochondrial functionality and integrity determination

MitoTracker Red CM-H2XRos and MitoTracker^®^ Deep Reed FM were used to assess mitochondria function and integrity. Briefly, in both cases cells were treated with 0.5, 1 and 2.5 μM C816 and C830 for 24h and with the same concentrations of C800 for 48 h. Culture medium was then removed and a prewarmed (37°C) solution containing 200 nM MitoTracker^®^ probe was added for mitochondria staining. Cells were incubated for 30 min at 37°C protected from the light. MitoTracker^®^ Red CM-H2XRos emission was measured using a Bio-Tek Synergy plate reader (exc 579 nm- em 599 nm) while MitoTracker^®^ Deep Reed FM-stained cells were mechanically removed by scraping, rinsed twice with fresh pre-warmed media and fixed with 4% paraformaldehyde (PFA) for 20 min at 4°C. Supernatants were finally discarded and pellets resuspended in PBS prior to analysis using a FACSCalibur™ cytometer. A total number of 10 000 events were acquired for each condition. Three experiments were performed.

### Real-time PCR

Cells were treated with 0.5, 1 and 2.5 μM C816, C830 and C800 for 24 h. Total RNA was obtained using the Aurum™ Total RNA Mini Kit following the manufacturer's instructions. RNA concentration and purity were determined using a Nanodrop™ 2000 spectophotometer (Thermo Fisher Scientific) and cDNA was synthesized using an oligo-dT and a RevertAid™ M-MuLV reverse transcriptase following the instructions provided by the manufacturer. Real-time PCR was performed using iTaq™ Universal SYBR^®^ Green Supermix in a StepOne™ real-time PCR system (Applied Biosystems) and data were analyzed with the StepOne™ Software (Applied Biosystems). Peptidylprolyl isomerase A gene (PPIA) was used as an internal normalization control and relative quantification between samples was performed using the ΔΔCt method. Each experimental condition was analyzed in triplicate.

### Caspase-3 activity

Control, 0.5, 1 and 2.5 μM C816, C830 and C800 treated cells, alone or in combination with 50 μM Pifithrin α, were harvested and rinsed twice with PBS before cellular lysis. Caspase-3 activity was determined using the EnzChek^®^ Caspase-3 Assay kit following the manufacturer's instructions.

### Zebrafish toxicity assessment and tumor xenograft assay

Adult zebrafish (WT) were fed and kept together at 28.5°C with a light/dark cycle of 14/10 h following The Zebrafish Book recommendations [45]. Embryos were obtained as massive spawning (non directed crossing) inside the aquarium for both, toxicity and xenograft assays, and were maintained in sterile dechlorinated tap water (SDTW).

The maximum non-toxic dose of C816 on zebrafish embryos was initially established by a toxicity assay conducted at 35°C in 96 well-plates for 96 h. Three different doses of C816, dissolved in 0,1 % DMSO, were evaluated on 48 hpf (hours post fecundation) embryos (10, 5 and 1 μM; n=8 per concentration). SDTW was included as negative control (n=24) and 3,4-dichloroaniline (DCA: 20, 10 and 4 mg/L; n=8 per concentration) was selected as positive control to check the sensitivity of the fish strain used following the recommendations for the zebrafish embryo toxicity test (OECD Test No. 236). All individuals exposed to 10 and 20 mg/L DCA died, and just 12.50% of those exposed to 4 mg/L survived but with important morphological alterations, corroborating the sensibility of the performed assay.

The zebrafish xenograft model was established by transplantation of stable fluorescent colorectal carcinoma cells expressing GFP protein (HCT-116-GFP) into 48 hpf zebrafish embryos. After manipulation animals were maintained in 96 well-plates at 35°C for 1 day to favor tumor cell proliferation and embryo recovering.

Experiments were carried out using a total number of 62 negative controls (non-injected) and 71 microinjected non-treated embryos, both maintained in SDTW. Different doses of C816 (0.5 μM, 1 μM, and 2 μM) were tested in 44, 40 and 25 individuals, respectively for 48 h. Another 40 embryos were also exposed to 500 μM 5′-fluoracile (5-FU) for the same period of time (48 h).

Six experiments were performed and both, tumor volumes and embryo survival rates, were annotated.

### Computerized image analysis

Tumor volumes were individually recorded for each embryo by image acquisition with a NIKON TE2000-3 confocal microscope set for GFP detection (Z-stacks of X-Y image planes) before and after treatment. Obtained images were then processed with the Image J software by collapsing the data from 3D into 2D using a z-projection (*sum projection*) prior to tumor integrated intensity determination. The ratios of pre-treatment fluorescence intensity to post-treatment tumor intensity were finally calculated for each individual to determine tumor size variations. The percentage of tumor regression was calculated as the percentage ratio of the difference between baseline fluorescence intensity and final fluorescence to the baseline intensity.

Following all experiments, zebrafish embryos were euthanized by an overdose of tricaine. Care, use and treatment of zebrafish were done under approval of the Santiago de Compostela University Bioethics Committee in compliance with Principles of Laboratory Animal Care of national laws.

### Statistics

The results were analyzed using the SIGMAPLOT^®^ software. For *in vitro* experiments One-way ANOVA was used to test for differences among groups and the Holm-Sidak multiple-range test was used for multiple comparisons between groups. Tumor development statistical analyses were calculated by the Mann-Whitney Rank Sum test. Significance was accepted at *p* < 0.05 in all cases.

## SUPPLEMENTARY FIGURES



## References

[1] Molinski TF, Dalisay DS, Lievens SL, Saludes JP (2009). Drug development from marine natural products. Nat Reviews Drug Discovery.

[2] Mayer AM, Glaser KB, Cuevas C, Jacobs RS, Kem W, Little RD, McIntosh JM, Newman DJ, Potts BC, Shuster DE (2010). The odyssey of marine pharmaceuticals: a current pipeline perspective. Trends in pharmacological sciences.

[3] Berlinck R, Braekman JC, Daloze D, Bruno I, Riccio R, Ferri S, Spampinato S, Speroni E (1993). Polycyclic guanidine alkaloids from the marine sponge Crambe crambe and Ca++ channel blocker activity of crambescidin 816. Journal of natural products.

[4] Berlinck R, Braekman JC, Daloze D, Hallenga K, Ottinger R, Bruno I, Riccio R (1990). Two new guanidine alkaloids from the mediterranean sponge crambe crambe. Tetrahedron letters.

[5] Jares-Erijman EA, Sakai R, Rinehart KL (1991). Crambescidins: new antiviral and cytotoxic compounds from the sponge Crambe crambe. The Journal of Organic Chemistry.

[6] Rubiolo JA, Ternon E, López-Alonso H, Thomas OP, Vega FV, Vieytes MR, Botana LM (2013). Crambescidin-816 acts as a fungicidal with more potency than crambescidin-800 and-830, inducing cell cycle arrest, increased cell size and apoptosis in Saccharomyces cerevisiae. Marine drugs.

[7] Suna H, Aoki S, Setiawan A, Kobayashi M (2007). Crambescidin 800, a pentacyclic guanidine alkaloid, protects a mouse hippocampal cell line against glutamate-induced oxidative stress. Journal of Natural Medicines.

[8] Lazaro JEH, Nitcheu J, Mahmoudi N, Ibana JA, Mangalindan GC, Black GP, Howard-Jones AG, Moore CG, Thomas DA, Mazier D (2006). Antimalarial activity of crambescidin 800 and synthetic analogues against liver and blood stage of Plasmodium sp. The Journal of antibiotics.

[9] Aoki S, Kong D, Matsui K, Kobayashi M (2004). Erythroid differentiation in K562 chronic myelogenous cells induced by crambescidin 800, a pentacyclic guanidine alkaloid. Anticancer research.

[10] Aron ZD, Pietraszkiewicz H, Overman LE, Valeriote F, Cuevas C (2004). Synthesis and anticancer activity of side chain analogs of the crambescidin alkaloids. Bioorganic & medicinal chemistry letters.

[11] Rubiolo J, López-Alonso H, Roel M, Vieytes M, Thomas O, Ternon E, Vega F, Botana L (2014). Mechanism of cytotoxic action of crambescidin-816 on human liver-derived tumour cells. British Journal of Pharmacoly.

[12] Veinotte CJ, Dellaire G, Berman JN (2014). Hooking the big one: the potential of zebrafish xenotransplantation to reform cancer drug screening in the genomic era. Disease models & mechanisms.

[13] Haldi M, Ton C, Seng W, McGrath P (2006). Human melanoma cells transplanted into zebrafish proliferate, migrate, produce melanin, form masses and stimulate angiogenesis in zebrafish. Angiogenesis.

[14] Konantz M, Balci TB, Hartwig UF, Dellaire G, André MC, Berman JN, Lengerke C (2012). Zebrafish xenografts as a tool for in vivo studies on human cancer. Annals of the New York Academy of Sciences.

[15] Stern HM, Zon LI (2003). Cancer genetics and drug discovery in the zebrafish. Nature Reviews Cancer.

[16] Pruvot B, Jacquel A, Droin N, Auberger P, Bouscary D, Tamburini J, Muller M, Fontenay M, Chluba J, Solary E (2011). Leukemic cell xenograft in zebrafish embryo for investigating drug efficacy. haematologica.

[17] Peterson RT, Link BA, Dowling JE, Schreiber SL (2000). Small molecule developmental screens reveal the logic and timing of vertebrate development. Proceedings of the National Academy of Sciences.

[18] Newmeyer DD, Ferguson-Miller S (2003). Mitochondria: releasing power for life and unleashing the machineries of death. Cell.

[19] Desagher S, Martinou J-C (2000). Mitochondria as the central control point of apoptosis. Trends in cell biology.

[20] Lakhani SA, Masud A, Kuida K, Porter GA, Booth CJ, Mehal WZ, Inayat I, Flavell RA (2006). Caspases 3 and 7: key mediators of mitochondrial events of apoptosis. Science.

[21] Zhao B, Li L, Wang L, Wang C-Y, Yu J, Guan K-L (2012). Cell detachment activates the Hippo pathway via cytoskeleton reorganization to induce anoikis. Genes & development.

[22] Schoenwaelder SM, Burridge K (1999). Bidirectional signaling between the cytoskeleton and integrins. Current opinion in cell biology.

[23] Wade Harper J, Adami GR, Wei N, Keyomarsi K, Elledge SJ (1993). The p21 Cdk-interacting protein Cip1 is a potent inhibitor of G1 cyclin-dependent kinases. Cell.

[24] Bates S, Bonetta L, MacAllan D, Parry D, Holder A, Dickson C, Peters G (1994). CDK6 (PLSTIRE) and CDK4 (PSK-J3) are a distinct subset of the cyclin-dependent kinases that associate with cyclin D1. Oncogene.

[25] Vidal A, Koff A (2000). Cell-cycle inhibitors: three families united by a common cause. Gene.

[26] El-Deiry WS, Harper JW, O'Connor PM, Velculescu VE, Canman CE, Jackman J, Pietenpol JA, Burrell M, Hill DE, Wang Y (1994). WAF1/CIP1 is induced in p53-mediated G1 arrest and apoptosis. Cancer research.

[27] Kastan MB, Zhan Q, El-Deiry WS, Carrier F, Jacks T, Walsh WV, Plunkett BS, Vogelstein B, Fornace AJ (1992). A mammalian cell cycle checkpoint pathway utilizing p53 and GADD45 is defective in ataxia-telangiectasia. Cell.

[28] Hermeking H, Lengauer C, Polyak K, He T-C, Zhang L, Thiagalingam S, Kinzler KW, Vogelstein B (1997). 14-3-3σIs a p53-Regulated Inhibitor of G2/M Progression. Molecular cell.

[29] Waldman T, Kinzler KW, Vogelstein B (1995). p21 is necessary for the p53-mediated G1 arrest in human cancer cells. Cancer research.

[30] Ouyang L, Shi Z, Zhao S, Wang FT, Zhou TT, Liu B, Bao JK (2012). Programmed cell death pathways in cancer: a review of apoptosis, autophagy and programmed necrosis. Cell Proliferation.

[31] Toshiyuki M, Reed JC (1995). Tumor suppressor p53 is a direct transcriptional activator of the human bax gene. Cell.

[32] Villunger A, Michalak EM, Coultas L, Müllauer F, Böck G, Ausserlechner MJ, Adams JM, Strasser A (2003). p53-and drug-induced apoptotic responses mediated by BH3-only proteins puma and noxa. Science.

[33] Grimberg A (2000). P53 and IGFBP-3: apoptosis and cancer protection. Molecular genetics and metabolism.

[34] Benchimol S (2001). p53-dependent pathways of apoptosis. Cell death and differentiation.

[35] Khosravi-Far R (2004). Death receptor signals to the mitochondria. Cancer Biology & Therapy.

[36] Assoian RK, Zhu X (1997). Cell anchorage and the cytoskeleton as partners in growth factor dependent cell cycle progression. Current opinion in cell biology.

[37] Pawlak G, Helfman DM (2001). Cytoskeletal changes in cell transformation and tumorigenesis. Current opinion in genetics & development.

[38] Geiger B, Bershadsky A, Pankov R, Yamada KM (2001). Transmembrane crosstalk between the extracellular matrix and the cytoskeleton. Nature Reviews Molecular Cell Biology.

[39] Grossmann J (2002). Molecular mechanisms of “detachment-induced apoptosis—Anoikis”. Apoptosis.

[40] Frisch SM, Screaton RA (2001). Anoikis mechanisms. Current opinion in cell biology.

[41] Assoian RK (1997). Anchorage-dependent cell cycle progression. The Journal of cell biology.

[42] Bos JL (1989). Ras oncogenes in human cancer: a review. Cancer research.

[43] Benvenuti S, Sartore-Bianchi A, Di Nicolantonio F, Zanon C, Moroni M, Veronese S, Siena S, Bardelli A (2007). Oncogenic activation of the RAS/RAF signaling pathway impairs the response of metastatic colorectal cancers to anti–epidermal growth factor receptor antibody therapies. Cancer research.

[44] Bondu S, Genta-Jouve G, Leirós M, Vale C, Guigonis J-M, Botana LM, Thomas OP (2012). Additional bioactive guanidine alkaloids from the Mediterranean sponge Crambe crambe. RSC Advances.

[45] Westerfield M (2000). The zebrafish book: a guide for the laboratory use of zebrafish.

