# Supplementary material for: Marine guanidine alkaloids crambescidins inhibit tumor growth and activate intrinsic apoptotic signaling inducing tumor regression in a colorectal carcinoma zebrafish xenograft model
Source: Oncotarget. 2016 Nov 4;7(50):83071–87. doi: 10.18632/oncotarget.13068 (PMC5347754; doi:10.18632/oncotarget.13068)
Supplement: Supplementary file 1 [file oncotarget-07-83071-s001.pdf]

Marine guanidine alkaloids crambescidins inhibit tumor growth and activate intrinsic apoptotic signaling inducing tumor regression in a colorectal carcinoma zebrafish xenograft model

SUPPLEMENTARY FIGURES

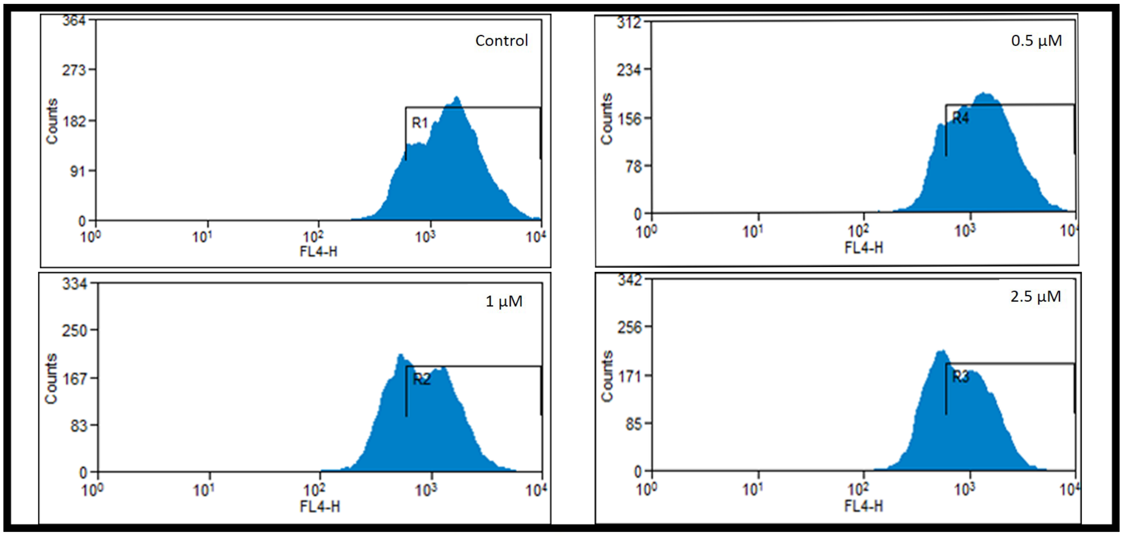

Supplementary Figure S1: Quantification of mitochondrial viability of control and 0.5 µM, 1 µM, and 2.5 µM C816-treated cells after 24 h as determined by flow cytometry analysis of Mitotracker® Deep Red-stained cells.

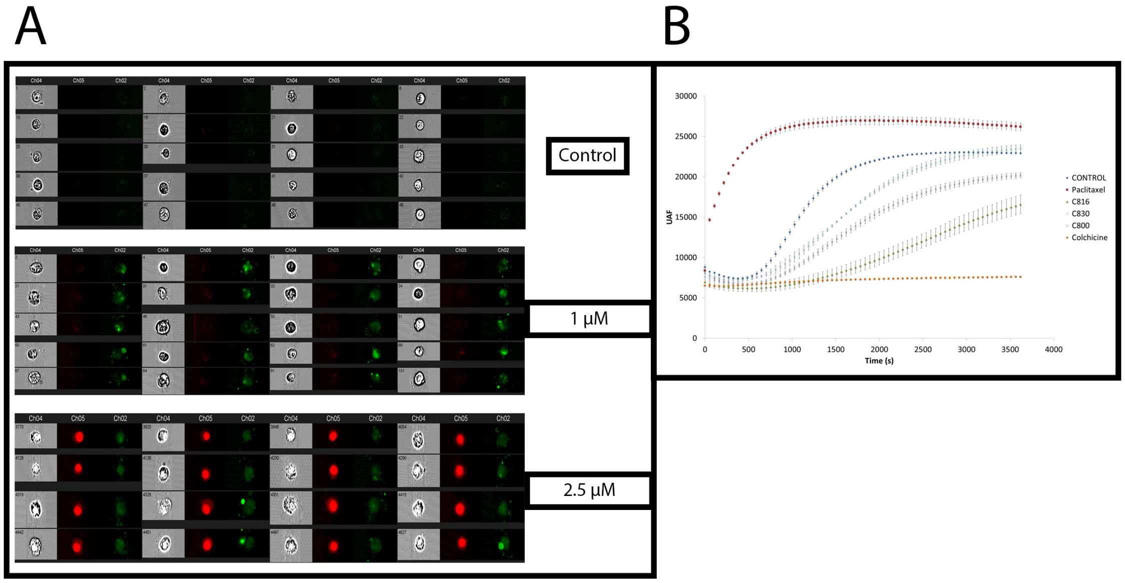

Supplementary Figure S2: A. Representative images of control and C816-treated cells (24h) as obtained by flow cytometry analysis using an lmageStream cytometer and Annexin V/IP. **B.** Tubulin polymerization was assayed in the presence of 10 µM paclitaxel (tubulin polymerization enhancer), 30 µM colchicine (tubulin polymerization inhibitor), 2.5 µM C800, 2.5 µM C830, 1 µM C816, or vehicle. Polymerization was determined espectrofluorimetrically during 1 h, with a measurement performed every 54 s. AUF: arbitrary fluorescence units.
